# Effective dose of remimazolam co-administered with remifentanil to facilitate I-gel insertion without neuromuscular blocking agents: an up-and-down sequential allocation trial

**DOI:** 10.1186/s12871-023-02041-z

**Published:** 2023-03-16

**Authors:** Juyeon Oh, Sung Yong Park, Ga Yun Lee, Ji Hyun Park, Han Bum Joe

**Affiliations:** 1 Department of Anesthesiology and Pain Medicine, Ajou University School of Medicine, 164 Worldcup-Ro, Yeongtong-Gu, Suwon, 16499 Republic of Korea; 2 Office of Biostatics, Medical Research Collaborating Center, Ajou Research Institute for Innovative Medicine, Ajou University Medical Center, Suwon, Republic of Korea

**Keywords:** General anesthesia, I-gel insertion, Remimazolam, Remifentanil, Neuromuscular blocking agents

## Abstract

**Background:**

Remimazolam is a new anesthetic drug developed and is an ultra-short-acting agent with rapid onset and offset. The pharmacology of this drug seems to be ideal for short surgeries eligible for I-gel insertion. Therefore, this study aimed to determine the optimal bolus dose of remimazolam for I-gel insertion when co-administered with remifentanil without neuromuscular blocking agents (NMBAs).

**Methods:**

Patients aged 19–65 years with American Society of Anesthesiologists physical status I or II scheduled for general anesthesia were enrolled. The first dose of remimazolam was 0.15 mg/kg and remifentanil was co-administered at an effect-site concentration (Ce) of 3.0 ng/mL. The dose of remimazolam for the following patient was decreased or increased by 0.05 mg/kg depending on the success or failure of I-gel insertion in the previous patient.

**Results:**

The remimazolam bolus dose required for successful I-gel insertion in 50% of adult patients using modified Dixon’s up-and-down method with remifentanil Ce 3.0 ng/mL and no NMBAs was 0.280 ± 0.048 mg/kg. Isotonic regression analysis showed that the 50% and 95% effective doses were 0.244 (83% confidence interval [CI] 0.213–0.313) mg/kg and 0.444 (95% CI 0.436–0.448) mg/kg, respectively. The mean time to loss of consciousness (Modified Observer’s Assessment of Alertness/Sedation score < 2) was 52.2 s. Three patients (12.0%) showed a reduction in systolic blood pressure of more than 30% from baseline.

**Conclusions:**

Selecting the appropriate dose of remimazolam/remifentanil without NMBAs makes it feasible to insert the I-gel.

**Trial registration:**

This study protocol was registered at http://cris.nih.go.kr (KCT0007801, 12th, October, 2022).

## Background

Supraglottic airway devices (SADs) are widely used for securing the airway and are known to be less invasive than endotracheal tubes. Consequently, compared to endotracheal tubes, SADs lead to lesser hemodynamic changes while inserting the devices and cause lesser irritation to the trachea, which can cause postoperative sore throat [1, 2]. I-gel, which was used in this study, is one type of SAD; as mentioned, it is less irritating to the larynx compared to other cuffed airway maintainers. Additionally, they are easy to insert and remove. Although SADs can be inserted without the use of neuromuscular blocking agents (NMBAs), a sufficient depth of anesthesia is necessary for their placement [3].

Propofol is the preferred drug for SAD insertion because of its ability to suppress the airway reflex [4]. However, it is difficult to completely block the airway reflex with propofol alone; therefore, additional opioids, such as remifentanil, are co-administered to facilitate insertion with minimal adverse hemodynamic disturbances [5–7].

Remimazolam is a new anesthetic drug developed and is an ultra-short-acting agent with a rapid onset and a short context-sensitive halftime of 6.8 ± 2.4 min (mean ± standard deviation, SD) allowing fast recovery [8]. Many previous studies have shown the non-inferiority of remimazolam to propofol in both procedural sedation and general anesthesia [9–11].

For short surgical procedures or outpatient anesthesia, the fast offset of NMBAs is a prime issue. Therefore, some authors recommend an induction regimen using propofol combined with a short-acting opioid without using NMBAs, as it provides a safe and fast recovery without residual effects of NMBAs [12]. As remimazolam allows a fast offset, it seems logical to use this advantage to perform general anesthesia by inserting an SAD without using NMBAs for short operations. However, to the best of our knowledge, the effective bolus dose of remimazolam for insertion of I-gel without NMBAs is not yet well known. Therefore, the present study aimed to determine the 50% effective dose (ED_50_) of remimazolam co-administered with remifentanil to facilitate I-gel insertion without using NMBAs.

## Methods

The study protocol was approved by the Institutional Review Board (AJOUIRB-IV-2022–360) of our institution and was registered at the Clinical Research Information Service (CRIS No. KCT0007801, 12/10/2022). This study was conducted at Ajou University Hospital (Suwon, Republic of Korea) between October 2022 and November 2022. All patients were provided with adequate information regarding the study and written informed consent was obtained from all patients. This study was performed in accordance with the principles of the Declaration of Helsinki.

### Patients

Patients aged 19–65 years with American Society of Anesthesiologists (ASA) physical status I or II scheduled for general anesthesia and eligible for I-gel insertion were enrolled in this study. The exclusion criteria were as follows: 1) body mass index > 30 kg/m^2^; 2) anticipated difficult airway; 3) anticipated difficult mask ventilation; 4) chronic obstructive pulmonary disease, asthma, pneumonia, active upper respiratory infection; 5) risk of aspiration; 6) medication known to interact with benzodiazepines such as insomnia drugs, proton-pump inhibitors, or certain antibiotics; 7) history of habitual use of benzodiazepines; 8) history of allergy to benzodiazepines or opioids; 9) history of substance abuse or addiction; and 10) pregnant or breastfeeding women.

### Anesthesia

No premedication was administered before induction, and standard monitors were applied as the patients arrived at the operating room. Non-invasive blood pressure, electrocardiography, pulse oximetry, and bispectral index (BIS) (A-2000™, Aspect Medical Systems, Newton, MA) were assessed. Prior to induction of anesthesia, each patient breathed spontaneously with 100% oxygen for preoxygenation.

Remimazolam was prepared in a syringe by a nurse (1 mg/mL), and the predetermined bolus dose of remimazolam was injected intravenously over few seconds by an anesthesiologist who calculated the induction dose of remimazolam. Simultaneously, remifentanil was infused using a total intravenous anesthesia pump (Orchestra® Base Primea; Fresenius Vial, Brezins, France) with an effect-site concentration (Ce) of 3.0 ng/mL. The other anesthesiologist, who was blinded to the dose of remimazolam, assessed the patient’s loss of consciousness (LOC). The LOC of the patient was measured using the Modified Observer’s Assessment of Alertness/Sedation (MOAA/S) score (Table 1).

**Table 1:** Modified Observer’s Assessment of Alertness/Sedation (MOAA/S) score

Score	Response
5	Subject responds readily to name spoken in normal tone
4	Lethargic response of subject to name spoken in normal tone
3	Subject responds only after name is called loudly and/or repeatedly
2	Subject responds only after mild prodding or shaking
1	Subject responds only after painful trapezius squeeze
0	Subject does not respond to painful trapezius squeeze

For successful insertion of the I-gel, the following conditions were achieved: 1) successful LOC (defined as MOAA/S < 2), 2) loss of spontaneous breathing, 3) Ce of remifentanil = 3.0 ng/mL, and 4) 150 s had passed after injecting the remimazolam bolus considering the peak effect time of remimazolam [8].

After these conditions were fulfilled, the anesthesiologist who checked the LOC inserted the I-gel. Successful I-gel insertion was defined as the smooth insertion of the device and symmetrical chest wall movement with a rectangular capnographic wave observed with manual ventilation. Smooth insertion comprised no involuntary movement of the body, no resistance to opening the patient’s mouth, and no cough or laryngospasm.

Only one attempt was made, and for safety and comfort of the failed patients, we immediately started remimazolam infusion at 1–2 mg/kg/h and top-up remifentanil Ce to 4.0 ng/mL and administered 20–30 mg of rocuronium intravenously. For successful patients, we started remimazolam infusion at 1–2 mg/kg/h to maintain the BIS value of 40–60, and decreased remifentanil infusion at 1.0–2.0 ng/mL of Ce before the surgery started.

Vital signs and BIS were recorded at baseline (T_0_) before induction of anesthesia, at LOC (T_1_), immediately after I-gel insertion (T_2_), 1 min after I-gel insertion (T_3_), 3 min after I-gel insertion (T_4_), 5 min after I-gel insertion (T_5_), and 10 min after I-gel insertion (T_6_). During the study period, if the patient’s mean arterial blood pressure (MAP) was < 65 mmHg or systolic arterial blood pressure (SBP) decreased by > 30% of the baseline, 4–8 mg of ephedrine was administered intravenously. If the heart rate (HR) dropped below 50 bpm, 0.5 mg atropine was injected.

The remimazolam dose in each patient was determined using Dixon’s up-and-down method. The initial remimazolam dose was deduced from a previous report that showed the mean (± SD) cumulative hypnotic dose of remimazolam was 0.17 ± 0.04 mg/kg and 0.29 ± 0.08 mg/kg in each 6 mg/kg/h and 12 mg/kg/h continuous remimazolam infusion [10]. Considering the synergistic effect of remifentanil and remimazolam and our clinical experience, we chose the initial remimazolam dose to be 0.15 mg/kg. The dose interval was selected based on our clinical experience [13] and the previous study’s SD [10]. As Dixon and Mood recommend the step size to lie between 0.5 and 2 times of the anticipated SD, we chose the dose interval as 0.05 mg/kg [14]. Therefore, the first patient received 0.15 mg/kg of remimazolam and when I-gel insertion was successful, the dose was decreased by 0.05 mg/kg in the next patient. When I-gel insertion was unsuccessful (failed), the dose was increased by 0.05 mg/kg in the next patient.

### Statistical analysis

At least six crossover pairs in the same direction and at least 20 patients are required for statistical analysis according to modified Dixon’s up-and-down method; therefore, patient enrollment continued until at least six success-to-failure pairs with 20 or more patients were reached [15–17]. The ED_50_ estimated using modified Dixon’s up-and-down method is the mean value of the independent crossover pairs.

We also analyzed our data using isotonic regression with a pooled adjacent violators algorithm (PAVA) and a bootstrapping approach to estimate the ED_50_ and ED_95_ of remimazolam along with confidence intervals (CIs).

Hemodynamic and BIS variables over time were analyzed using one-way repeated measure analysis of variance with a Bonferroni correction. Paired sample t-tests were used to compare baseline hemodynamic and BIS variables with other periods. Analyses of these results were performed for patients in whom I-gel insertion was successful.

LOC time was compared between the success and failure groups using a two-sample t-test. Analysis of adverse events between the success and failure groups was performed using Fisher’s test. All values are expressed as mean ± SD or number of patients. Statistical analyses were performed using R (version 4.05; R Foundation for Statistical Computing, Vienna, Austria) and a *P* value < 0.05 was considered statistically significant.

## Results

**Fig. 1:**
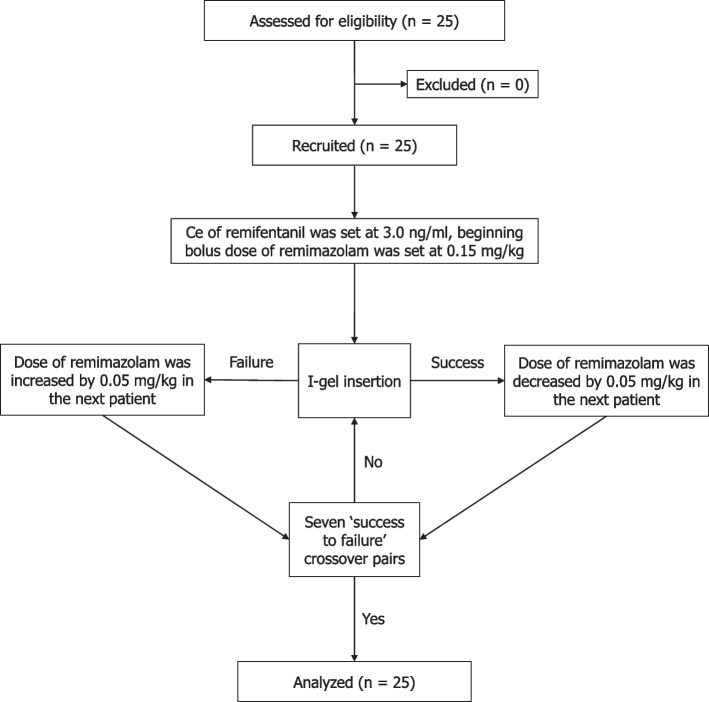
Flow diagram for the Dixon’s up-and-down method

Twenty-five patients were enrolled in this study (Fig. 1). The demographic characteristics of the patients are presented in Table 2.

**Table 2:** Demographic data and time to LOC

Parameters	Success (*N* = 11)	Fail (*N* = 14)	Total (*N* = 25)
Age (years)	48.3 ± 11.9	44.0 ± 13.2	45.9 ± 12.6
Sex (M/F)	5/6	8/6	13/12
Weight (kg)	64.8 ± 10.7	65.4 ± 13.4	65.1 ± 12.0
Height (cm)	163.9 ± 7.8	166.2 ± 7.4	165.2 ± 7.5
BMI (kg/m^2^)	24.0 ± 2.8	23.5 ± 3.4	23.8 ± 3.1
ASA PS (I/II)	6/5	10/4	16/9
Time to LOC (s)	50.7 ± 14.6	53.4 ± 13.3	52.2 ± 13.7

Values are presented as mean ± standard deviation or number. There was no significant difference between the success and failure groups

*LOC* Loss of consciousness, *BMI* Body mass index, *ASA PS* American Society of Anesthesiologists physical status

**Fig. 2:**
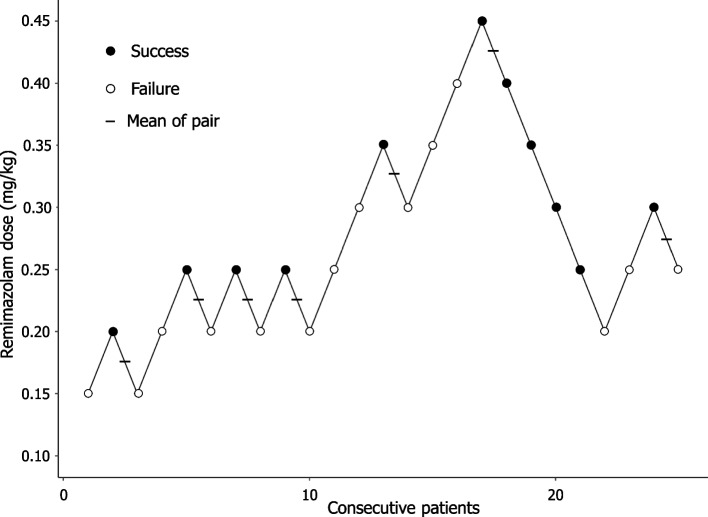
The responses of 25 consecutive patients to I-gel insertion. A successful insertion dose is denoted by a solid circle; a failed insertion dose is denoted by an open circle; horizontal bars represent crossover midpoints (success-to-failure)

**Fig. 3:**
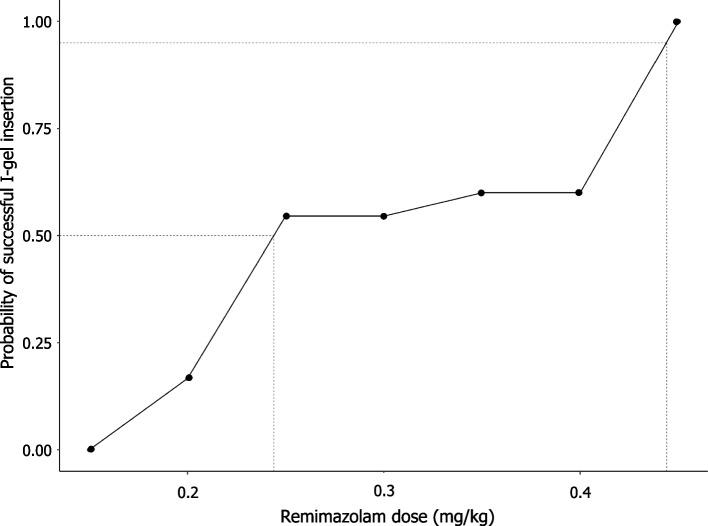
The pooled adjacent violators algorithm (PAVA) probability of successful I-gel insertion of remimazolam dose co-administered with remifentanil at an effect-site concentration of 3 ng/mL. The 50% and 95% effective doses were 0.244 (83% CI 0.213–0.313) mg/kg and 0.444 (95% CI 0.436–0.448) mg/kg, respectively

Figure 2 shows the plots of the dose of remimazolam associated with the success or failure of I-gel insertion for each consecutive patient. The ED_50_ calculated using modified Dixon’s up-and-down method from seven crossover pairs was 0.280 ± 0.048 mg/kg [14]. From the isotonic regression analysis, ED_50_ and ED_95_ were 0.244 mg/kg (83% CI 0.213–0.313) and 0.444 mg/kg (95% CI 0.436–0.448), respectively (Table 3). Figure 3 depicts the isotonic regression calculated using PAVA and the bootstrapping approach.

**Table 3:** Dose of remimazolam needed for insertion of I-gel

	Patients (*N* = 25)
Dixon’s up-and-down method	
ED_50_ (mg/kg)	0.280 ± 0.048
Isotonic regression method	
ED_50_ (mg/kg)	0.244 (0.213–0.313)
ED_95_ (mg/kg)	0.444 (0.436–0.448)

Data from Dixon’s up-and-down method are presented as the mean ± standard deviation. Data from the isotonic regression method were the ED_50_ (83% CI) and ED_95_ (95% CI)

*ED*_*50*_ Effective dose in 50% of the sample, *ED*_*95*_ Effective dose in 95% of the sample, *CI* Confidence interval

**Fig. 4:**
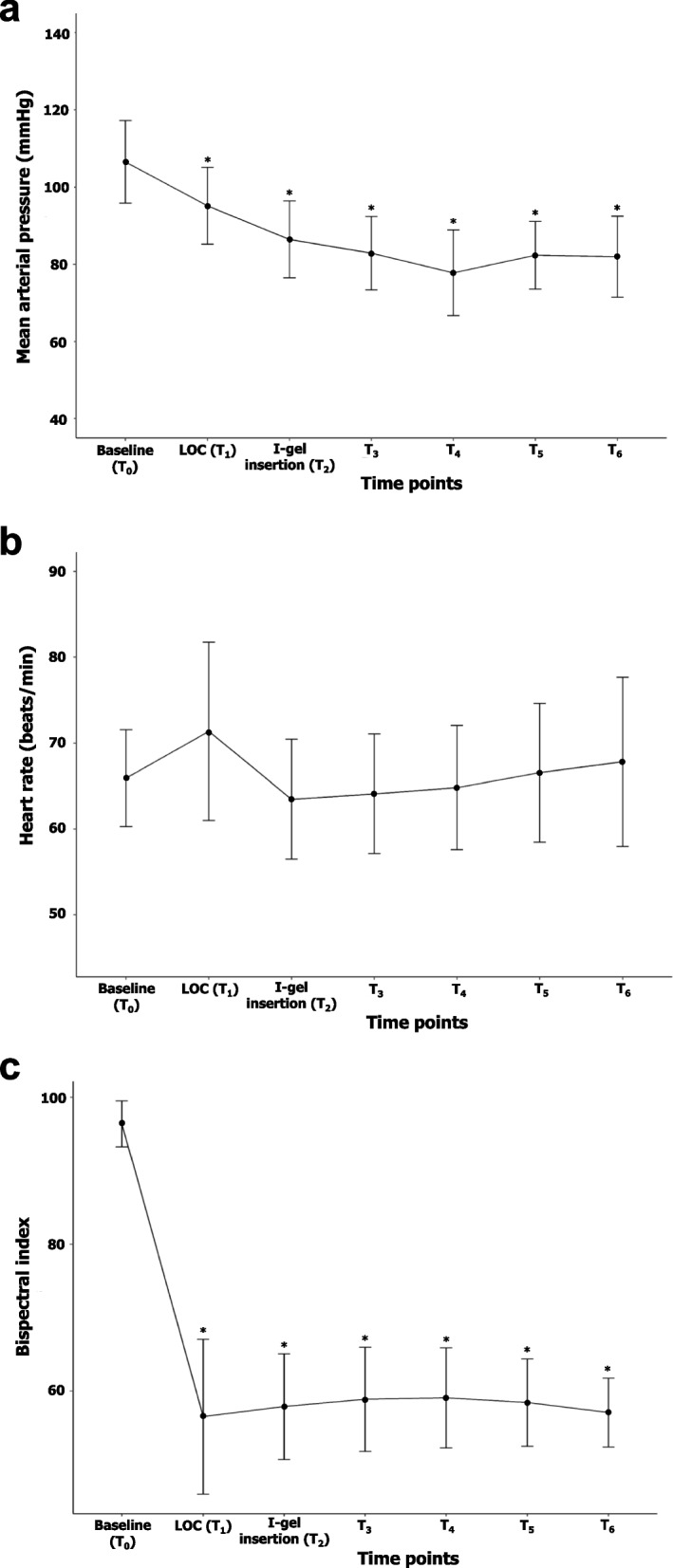
Changes in mean arterial pressure (**a**), heart rate (**b**), and bispectral index (**c**) during study period. Data are expressed as the mean ± standard deviation. T_0_, baseline; T_1_, loss of consciousness; T_2_, immediately after I-gel insertion; T_3_, 1 min. after I-gel insertion; T_4_, 3 min. after I-gel insertion; T_5_, 5 min. after I-gel insertion; T_6_, 10 min. after I-gel insertion. *P < 0.05 compared to baseline

The hemodynamic and BIS changes were compared between the baseline and each point in time for data collection (Fig. 4). MAP significantly decreased throughout the study period compared with that at baseline (Fig. 4a). The maximum drop in SBP compared to baseline was 25.5 ± 13.8%, and the specific period for the largest drop in blood pressure was T_4_, which was 3 min after I-gel insertion. The HR increased the most at T_1_ (LOC time) compared to the baseline and then showed a decreasing trend, but this was not statistically significant (Fig. 4b). The BIS values decreased significantly and remained at approximately 60 during the study period (Fig. 4c).

The LOC time of all patients was 52.2 ± 13.7 s; LOC time was not significantly different between the success and failure groups (Table 2). During remimazolam induction, three patients (12.0%) showed hypotension (SBP decreased by more than 30% from baseline) and received 8 mg of ephedrine. There were two hiccup events in the failure group, and the main causes of unsuccessful I-gel insertion were limb movement, facial frowning, resistance to mouth opening, and resistance to I-gel insertion.

## Discussion

Using modified Dixon’s up-and-down method, we found that the remimazolam bolus dose needed for successful I-gel insertion in 50% of adult patients was 0.280 ± 0.048 mg/kg when co-administered with Ce 3.0 ng/mL of remifentanil without NMBAs. Moreover, remimazolam bolus induction resulted in a mean LOC time (MOAA/S < 2) of 52.2 s. This study showed that remimazolam bolus can be used in combination with remifentanil for I-gel insertion with rapid induction when patients need to recover without the concern of residual neuromuscular blockade.

Remifentanil is a short-acting μ-receptor agonist and, similar to remimazolam, it produces a fast onset and has the advantage of quick degradation by plasma or tissue esterase, showing high clearance and a short context-sensitive half-life [18, 19]. This advantage of remifentanil is beneficial to operations that require intense analgesia for a short period or in cases that require continuous analgesic infusion, even in short surgeries. In fact, narcotic analgesics show synergistic effects when co-administered with propofol or midazolam [20–22]. A study investigating remimazolam/remifentanil in cynomolgus monkeys revealed a high degree of synergism between remimazolam and remifentanil [23]. Synergistic interactions between drugs are clinically useful because they can reduce the potential side effects of each drug by allowing individual drugs to be used in smaller doses. However, on the other hand, when the dose is titrated without considering the synergistic effect of drugs, side effects such as severe cardio-respiratory depression may also appear stronger; therefore, careful selection considering the interaction between drugs is necessary when determining the dose [6, 23]. However, the appropriate remimazolam dose required to insert the I-gel when remifentanil is used together with an opioid agent remains unclear. This study aimed to identify the optimal dose of remimazolam with fewer side effects.

As mentioned previously, propofol has been extensively used for SAD insertion because it strongly inhibits airway reflex. Numerous SAD studies have found an appropriate dose of remifentanil co-administered with propofol to facilitate the device [24, 25]. The optimal Ce for remifentanil was mostly around 3.0–5.0 ng/mL; therefore, we set the remifentanil dose at 3.0 ng/mL in this study [7, 24, 26]. The use of opioids blunts hemodynamic responses to painful stimuli and reduces the dose for other anesthetic agents. Considering the synergistic effect, we selected the initial dose of remimazolam (0.15 mg/kg), which was lower than the least successful mean hypnotic dose of remimazolam co-administered with remifentanil infusion in a previous study [10]. However, in this study, 3 of 25 patients (12.0%) showed hypotension, in which SBP decreased by more than 30% from the baseline. In a previous prospective observational study of I-gel insertion with 6 mg/kg/h of remimazolam co-administered with a Ce of 4.0 ng/mL remifentanil, the incidence of hypotension (21.6%) was greater than that in our study [27]. Higher concentrations of remifentanil may have affected the incidence of hypotension. Moreover, in a recent study, the 95% effective Ce of remifentanil for I-gel insertion under remimazolam induction of 12 mg/kg/h was 2.07 (95% CI 1.94–2.87) ng/mL and hypotension did not occur [28]. Therefore, titrating the Ce of remifentanil to less than 3.0 ng/mL might be beneficial for maintaining tighter hemodynamic stability.

When a new drug is developed, researchers continue to find efficient methods to improve safety, efficacy, onset, and recovery profiles, while minimizing side effects [29]. Innovations in drug delivery have contributed to improvements in anesthesia. Many intravenous sedatives such as thiopental, propofol, ketamine, and midazolam are usually administered in a bolus shot manner, which provides rapid induction. In a previous study, the average time to LOC by continuous remimazolam infusion of 6 and 12 mg/kg/h co-administered with remifentanil infusion between 0.25 and 0.5 µg/kg/min was 102 and 88.7 s, respectively [10]. The prospective observational study mentioned above showed that the mean time of LOC was 63 (interquartile range 54.0–76.8) s [27]. In the present study, the mean time to LOC (MOAA/S < 2) that was measured from the beginning of the bolus administration was 52.2 ± 13.7 s, which was shorter than the two previous studies. According to our results, we can gain the advantage of the fast onset time of remimazolam using a bolus injection method. Furthermore, flumazenil can rapidly reverse the hypnotic effect of remimazolam. Therefore, these methods can conveniently reduce the induction and recovery times of remimazolam.

Clinically, a BIS value of 40–60 indicates a safety profile of sedation depth under general anesthesia. However, the databases used to develop the BIS were based on propofol and did not include electroencephalogram (EEG) data from benzodiazepine [30]. Therefore, the depth of sedation and BIS are weakly correlated with midazolam compared to propofol [31]. A previous study revealed that the EEG changes after an intravenous bolus of midazolam were positively correlated with beta activation, and the average BIS value remained over 60 after induction [32]. In this study, we observed the BIS for several patients who were above 60, but the MOAA/S score remained zero. In addition, there were no recalls for any of the patients. Thus, the effect of remimazolam on EEG changes needs to be fully clarified, and the appropriate range of the EEG index for remimazolam should be more demonstrated later.

This study has several limitations. First, the remimazolam requirement for SAD can differ according to sex and age [33, 34]. In particular, a deeper sedation level was found in men than in women after administration of the same dose of midazolam [34]. Also, it is known that elderly patients usually need titrated doses of anesthetic drugs to minimize untoward cardio-respiratory events [34]. There is an obvious need for more research to determine the effects of sex and age on dose requirements. Second, this study was conducted only during the induction period. Patient profiles were collected for only 10 min. Therefore, recovery-related parameters should be further evaluated. Third, because the Ce of remifentanil was fixed in this study, the remimazolam ED with other remifentanil concentrations was unknown.

## Conclusion

The remimazolam bolus dose for successful I-gel insertion in 50% of adult patients with Ce 3.0 ng/mL of remifentanil and without NMBAs was 0.280 ± 0.048 mg/kg. From the isotonic regression analysis, ED_50_ and ED_95_ were 0.244 (83% CI 0.213–0.313) mg/kg and 0.444 (95% CI 0.436–0.448) mg/kg, respectively. Further studies to find more optimal combinations of remifentanil and remimazolam doses for I-gel insertion to ensure tighter hemodynamic stability will contribute to the safe use of remimazolam.

## Data Availability

The datasets generated and analyzed during the present study are available from the corresponding author on reasonable request.

## Abbreviations

ASA: American Society of Anesthesiologists
BIS: Bispectral index
Ce: Effect-site concentration
CI: Confidence interval
ED: Effective dose
EEG: Electroencephalography
HR: Heart rate
LOC: Loss of consciousness
MAP: Mean arterial pressure
MOAA/S: Modified Observer’s Assessment of Alertness/Sedation
NMBA: Neuromuscular blocking agent
PAVA: Pooled adjacent violators algorithm
SAD: Supraglottic airway device
SBP: Systolic blood pressure
SD: Standard deviation

## References

[1] Pennant JH, White PF (1993). The laryngeal mask airway Its uses in anesthesiology. Anesthesiology.

[2] Singh A, Bhalotra AR, Anand R (2018). A comparative evaluation of ProSeal laryngeal mask airway, I-gel and Supreme laryngeal mask airway in adult patients undergoing elective surgery: A randomised trial. Indian J Anaesth.

[3] Kong M, Li B, Tian Y (2016). Laryngeal mask airway without muscle relaxant in femoral head replacement in elderly patients. Exp Ther Med.

[4] Molloy ME, Buggy DJ, Scanlon P (1999). Propofol or sevoflurane for laryngeal mask airway insertion. Can J Anaesth.

[5] Bouillon TW, Bruhn J, Radulescu L, Andresen C, Shafer TJ, Cohane C (2004). Pharmacodynamic interaction between propofol and remifentanil regarding hypnosis, tolerance of laryngoscopy, bispectral index, and electroencephalographic approximate entropy. Anesthesiology.

[6] Hendrickx JF, Eger EI, Sonner JM, Shafer SL (2008). Is synergy the rule? A review of anesthetic interactions producing hypnosis and immobility. Anesth Analg.

[7] Zaballos M, Bastida E, Agusti S, Portas M, Jimenez C, Lopez-Gil M (2015). Effect-site concentration of propofol required for LMA-Supreme insertion with and without remifentanil: a randomized controlled trial. BMC Anesthesiol.

[8] Schuttler J, Eisenried A, Lerch M, Fechner J, Jeleazcov C, Ihmsen H (2020). Pharmacokinetics and Pharmacodynamics of Remimazolam (CNS 7056) after Continuous Infusion in Healthy Male Volunteers: Part I Pharmacokinetics and Clinical Pharmacodynamics. Anesthesiology.

[9] Chen S, Wang J, Xu X, Huang Y, Xue S, Wu A (2020). The efficacy and safety of remimazolam tosylate versus propofol in patients undergoing colonoscopy: a multicentered, randomized, positive-controlled, phase III clinical trial. Am J Transl Res.

[10] Doi M, Morita K, Takeda J, Sakamoto A, Yamakage M, Suzuki T (2020). Efficacy and safety of remimazolam versus propofol for general anesthesia: a multicenter, single-blind, randomized, parallel-group, phase IIb/III trial. J Anesth.

[11] Dai G, Pei L, Duan F, Liao M, Zhang Y, Zhu M (2021). Safety and efficacy of remimazolam compared with propofol in induction of general anesthesia. Minerva Anestesiol.

[12] Bettelli G (2006). Which muscle relaxants should be used in day surgery and when. Curr Opin Anaesthesiol.

[13] Oh J, Park SY, Lee SY, Song JY, Lee GY, Park JH (2022). Determination of the 95% effective dose of remimazolam to achieve loss of consciousness during anesthesia induction in different age groups. Korean J Anesthsiol.

[14] Dixon W.J, Mood A.M (1948). A method for Obtaining and Analyzing Sensitivity Data. J American Statistical Association.

[15] Paul M, Fisher DM (2001). Are estimates of MAC reliable?. Anesthesiology.

[16] Gorges M, Zhou G, Brant R, Ansermino JM (2017). Sequential allocation trial design in anesthesia: an introduction to methods, modeling, and clinical applications. Paediatr Anaesth.

[17] Dixon WJ (1991). Staircase bioassay: the up-and-down method. Nuerosci Biobehav Rev.

[18] Rosow CE (1999). An overview of remifentanil. Anesth Analg.

[19] Egan TD (1995). Remifentanil pharmacokinetics and pharmacodynamics A preliminary appraisal. Clin Pharmacokinet.

[20] Scott HB, Choi SW, Wong GT, Irwin MG (2017). The effect of remifentanil on propofol requirements to achieve loss of response to command vs. loss of response to pain. Anaesthesia.

[21] Avramov MN, Smith I, White PF (1996). Interactions between midazolam and remifentanil during monitored anesthesia care. Anesthesiology.

[22] Koh JC, Park J, Kim NY, You AH, Ko SH, Han DW (2017). Effects of remifentanil with or without midazolam pretreatment on the 95% effective dose of propofol for loss of consciousness during induction: A randomized, clinical trial. Medicine (Baltimore).

[23] Kops MS, Pesic M, Petersen KU, Schmalix WA, Stohr T (2021). Impact of concurrent remifentanil on the sedative effects of remimazolam, midazolam and propofol in cynomolgus monkeys. Eur J Pharmacol.

[24] Kim MK, Lee JW, Jang DJ, Shin OY, Nam SB (2009). Effect-site concentration of remifentanil for laryngeal mask airway insertion during target-controlled infusion of propofol. Anaesthesia.

[25] Grewal K, Samsoon G (2001). Facilitation of laryngeal mask airway insertion: effects of remifentanil administered before induction with target-controlled propofol infusion. Anaesthesia.

[26] Meng W, Kang F, Dong M, Wang S, Han M, Huang X (2022). Remifentanil requirement for i-gel insertion is reduced in male patients with Parkinson's disease undergoing deep brain stimulator implantation: an up-and-down sequential allocation trial. BMC Anesthesiol.

[27] Park I, Cho M, Nam SW, Hwang JW, Do SH, Na HS (2022). Total intravenous anesthesia induced and maintained by a combination of remimazolam and remifentanil without a neuromuscular blocking agent: a prospective, observational pilot study. BMC Anesthesiol.

[28] Choi JJ, Jung WS, Chang YJ, Yoo S, Kwak HJ. Effective concentration of remifentanil for successful i-gel insertion during remimazolam induction. Korean J Anesthesiol. 2022. 10.4097/kja.22606. 10.4097/kja.22606 PMC10244610 36314044

[29] Mahmoud M, Mason KP. Recent advances in intravenous anesthesia and anesthetics. F1000Res. 2018;7. 10.12688/f1000research.13357.1. 10.12688/f1000research.13357.1 PMC5911929 29755731

[30] Kim KM (2022). Remimazolam: pharmacological characteristics and clinical applications in anesthesiology. Anesth Pain Med (Seoul).

[31] Ibrahim AE, Taraday JK, Kharasch ED (2001). Bispectral index monitoring during sedation with sevoflurane, midazolam, and propofol. Anesthesiology.

[32] Miyake W, Oda Y, Ikeda Y, Hagihira S, Iwaki H, Asada A (2010). Electroencephalographic response following midazolam-induced general anesthesia: relationship to plasma and effect-site midazolam concentrations. J Anesth.

[33] Pleym H, Spigset O, Kharasch ED, Dale O (2003). Gender differences in drug effects: implications for anesthesiologists. Acta Anaesthesiol Scand.

[34] Sun GC, Hsu MC, Chia YY, Chen PY, Shaw FZ (2008). Effects of age and gender on intravenous midazolam premedication: a randomized double-blind study. Br J Anaesth.

